# Improving Contact Prediction along Three Dimensions

**DOI:** 10.1371/journal.pcbi.1003847

**Published:** 2014-10-09

**Authors:** Christoph Feinauer, Marcin J. Skwark, Andrea Pagnani, Erik Aurell

**Affiliations:** 1 DISAT and Center for Computational Sciences, Politecnico Torino, Torino, Italy; 2 Department of Information and Computer Science, Aalto University, Aalto, Finland; 3 Aalto Science Institute (AScI), Aalto University, Aalto, Finland; 4 Human Genetics Foundation-Torino, Molecular Biotechnology Center, Torino, Italy; 5 Department of Computational Biology, Royal Institute of Technology, AlbaNova University Centre, Stockholm, Sweden; Fox Chase Cancer Center, United States of America

## Abstract

Correlation patterns in multiple sequence alignments of homologous proteins can be exploited to infer information on the three-dimensional structure of their members. The typical pipeline to address this task, which we in this paper refer to as the *three dimensions of contact prediction*, is to (i) filter and align the raw sequence data representing the evolutionarily related proteins; (ii) choose a predictive model to describe a sequence alignment; (iii) infer the model parameters and interpret them in terms of structural properties, such as an accurate contact map. We show here that all three dimensions are important for overall prediction success. In particular, we show that it is possible to improve significantly along the second dimension by going beyond the pair-wise Potts models from statistical physics, which have hitherto been the focus of the field. These (simple) extensions are motivated by multiple sequence alignments often containing long stretches of gaps which, as a data feature, would be rather untypical for independent samples drawn from a Potts model. Using a large test set of proteins we show that the combined improvements along the three dimensions are as large as any reported to date.

## Author Summary

Proteins are large molecules that living cells make by stringing together building blocks called amino acids or peptides, following their blue-prints in the DNA. Freshly made proteins are typically long, structure-less chains of peptides, but shortly afterwards most of them fold into characteristic structures. Proteins execute many functions in the cell, for which they need to have the right structure, which is therefore very important in determining what the proteins can do. The structure of a protein can be determined by X-ray diffraction and other experimental approaches which are all, to this day, somewhat labor-intensive and difficult. On the other hand, the order of the peptides in a protein can be read off from the DNA blue-print, and such protein sequences are today routinely produced in large numbers. In this paper we show that many similar protein sequences can be used to find information about the structure. The basic approach is to construct a probabilistic model for sequence variability, and then to use the parameters of that model to predict structure in three-dimensional space. The main technical novelty compared to previous contributions in the same general direction is that we use models more directly matched to the data.

## Introduction

The large majority of cellular mechanisms are executed and controlled by the coordinated action of thousands of proteins, whose biological function is strongly connected to their three-dimensional (3D) arrangement. As shown by Anfinsen almost 40 years ago [1], the native three-dimensional structure and function of any given protein is unambiguously encoded by its amino acid sequence. Despite many years of intensive work in the field, and many partial successes, the problem of predicting structural properties of a protein from sequence information alone is still to be considered as an open problem.

Recent years have seen a staggering increase in the amount of available protein sequence data, which can be attributed to the developments in the sequencing technologies. Currently, sequences of more than 80 million proteins are known, which is a figure that continues growing by over 50% yearly [2]. This, coupled with advances in sequence homology detection methods [3]–[5], allows for construction of accurate multiple sequence alignments (MSA), capable of capturing the evolutionary history of proteins of interest. As a result of the trade-off between the evolutionary drift and the constraint imposed by biological function, proteins comprising such a multiple sequence alignment are generally characterized by: (i) a considerable sequence variation, (ii) a striking similarity between their 3D structures. In particular, the evolutionary pressure to conserve structure suggests that residues in spatial proximity should exhibit patterns of correlated amino acid substitutions in these multiple sequence alignments.

The approach of using co-evolutionary information encoded in the MSA of homologous proteins to predict structural features of its members was proposed long ago [6]–[11] (see also [12], [13] for recent reviews on the subject). The last five years have witnessed a renewed interest in the problem: after a first wave of works inspired by statistical physics based on Bayesian methods [14], [15], or on different mean-field approximations to a maximum-entropy model [16], [17], a burst of scientific activity produced new and increasingly accurate global inference methods [18]–[24]. Apart from inferring structural properties for single protein domains, co-evolutionary methods provide reliable predictions for: (i) inter-chain structural organization [17], (ii) specificity and partner identification in protein-protein interaction in bacterial signal transduction system [15], [25], (iii) essential residue-residue contacts to determine native 3D structures [26]–[28].

The basis of all these computational methods is the idea of global statistical inference. The global approach has the advantage that it is able to disentangle direct from indirect couplings between residues. By modeling the whole data set at once, and not only pairs of residues independently, it is, for example, possible to identify a case in which high correlation between two residues is the indirect consequence of both being directly correlated to a third variable.

Methods that address this problem are collected under the umbrella term of *Direct Coupling Analysis* (DCA). Some methods used so far are (i) the message passing based DCA (mpDCA) [16] and the mean-field DCA (mfDCA) [17], (ii) sparse inverse covariance methods (PSICOV) [20], (iii) pseudo-likelihood based optimization [18], [22], [23]. The techniques proposed in (iii), and in particular the *plmDCA* algorithm [22], [24], seem to achieve the most accurate predictions so far, when validated against experimentally determined protein structures. Nonetheless, plmDCA shows systematic errors that can be traced back to certain intrinsic characteristics of MSAs, such as the existence of repeated gap stretches in specific parts of the alignment. This phenomenon reflects the tendency of homologous proteins to include large-scale modular gene rearrangements in their phylogenetic evolution, as well as point insertions/deletions. As an empirical way to describe such complex rearrangements, sequence alignment methods typically use a form of substitution matrix to assign scores to amino acid matches and a gap penalty for matching an amino acid in one sequence and a gap in the other. In either case, the most widely utilized gap-penalty schemes assign a large cost to open a gap and a smaller one to extend a gap, so that the overall penalty *Q* of creating a stretch of gaps of length *l* is *Q*(*l*) = *a*+*b*(*l*−1), where typically *a*∼−10 and *b*∼−2 [29]. This introduces an intrinsic asymmetry between gaps and amino acids, where subsequences consisting only of the gap variable are much more likely to occur in an MSA than subsequences of one and the same amino acid.

In this work we highlight that contact prediction can be improved in three different ways, or *dimensions*, all important for overall success and accuracy. The first dimension is **Data**; it matters which MSA one uses as input to a DCA scheme. Continuing recent work of one of us [30] we show that in a large test data set MSAs built on HHblits alignments give more useful information than MSAs derived from the Pfam protein families database. This conclusion is perhaps not surprising, as the Pfam database was not constructed with potential applications to DCA in mind, but is practically important if DCA is to reach its full potential. The second dimension is **Model**; it matters which global model one tries to learn from an MSA, and it is possible to systematically improve upon the pairwise interaction models, or Potts models, which have hitherto been the focus of the field. This we show starting from the empirical observation that several DCA methods typically produce high-ranking false positives in parts of an alignment rich in gaps, and the simple fact that any subsequence of one of the same variable has low sequence entropy, and is thus unlikely to occur in random samples drawn from a Potts model, unless its model parameters take special values, *i.e.* unless at least some of them are quite large. We therefore enhance the Potts model by including terms depending on gaps of any length, much in the spirit of a simplified model for protein folding proposed long ago [31]. In this way we are able to effectively reduce the false positive rate in gap-rich regions of the MSA over a large test data set of diverse proteins. The third dimension is **Method**. It is well known that DCA by learning a Potts model describing an MSA by exactly maximizing a likelihood function is computationally unfeasible for realistic protein sizes. Most DCA methods can therefore be seen as circumventing this fact, either by approximating the likelihood function, or by using a different (weaker) learning criterion. Here, we show that pseudo-likelihood based optimization methods, which have demonstrated the best performance among standalone methods, have the additional advantage of being flexible and easily adaptable to learning other *models*. This we show by including terms depending on gaps of any length in the score function optimized in the recently developed *asymmetric* version of the plmDCA algorithm [22], [24] resulting in a method we denote gplmDCA. We show as well, that improvement achieved by introduction of gap terms can be attained also by a modification to the scoring of inferred matrices (plmDCA20).

Important recent developments, not touched upon in the present work, are combining two or more DCA methods and/or incorporating supplementary information in a prediction process, as done in [30] and [23]. One motivation is that it is theoretically interesting by itself to see how much useful information can be learned by simply starting from the data, proposing a model, and then learning the model more or less well from the data; a second motivation is computational speed, as a stand-alone method is (typically) much faster than meta-predictors. A pragmatic motivation for this choice is that any meta-predictor is based on combining stand-alone methods. Hence, improving stand-alone methods gives scope for further improvements of the meta-predictors. Indeed, we believe that the method developed here should allow for further improvements to the methods of [30] and [23]; this we leave however for future work.

## Results

### We have developed a new, fast DCA method by extending the Potts model with gap parameters

The new method *gap-enhanced pseudo maximum-likelihood direct contact analysis* (gplmDCA) uses as underlying inference engine the recent *asymmetric pseudo maximum-likelihood* [24] augmented by gap parameters, as described in Methods. The added gap parameters have the same status as the other parameters of the model, and the inference task posed by gplmDCA is therefore formally the same as in plmDCA. The number of additional parameters is less than 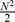, with *N* being the length of a alignment, a small fraction of the number of parameters in Potts model based DCA. We have found that the computing time our new method gplmDCA is almost indistinguishable from the asymmetric version of plmDCA [24].

**Figure 1:**
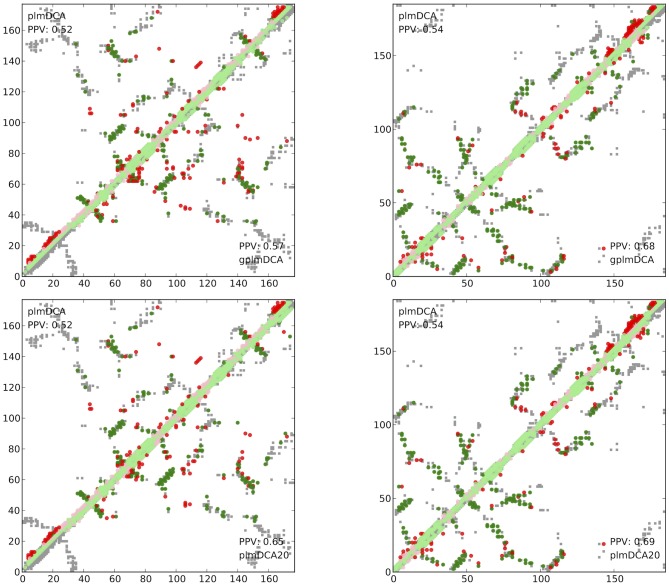
Examples of qualitative contact prediction improvement. Gray squares: contacts observed in crystal structure, Ovals: predicted contacts (green: correctly predicted, red: incorrectly predicted). Predicted very short-range contacts (not considered in the assessment) are drawn in pale colors.Top row: comparison of plmDCA and gplmDCA, bottom row: plmDCA and plmDCA20. Left panels: contact prediction maps built by plmDCA and gplmDCA/plmDCA20 using protein sequences homologous to 1JFU:A as explained in Methods. For this protein plmDCA predicts a number of strong couplings at both the N-terminus and the C-terminus, which arise from the high sequence variability at both ends of proteins homologous to 1JFU:A and the many gaps in the multiple sequence alignments at these positions. In gplmDCA these gaps lead to adjustment of gap parameters and not to contact predictions, in plmDCA20 these couplings are not included in contact scoring, leading to an analogous effect. Right panels: analogous results using protein sequences homologous to 1ATZ where gplmDCA and plmDCA20 remove strong spurious couplings at the C-terminus.

This introduction of gap parameters significantly alleviates a well-known negative trait of plmDCA – the presence of gap-induced artifacts in many contact maps. The reduction of strong, but spurious couplings in the inference process allows for the detection of other couplings, improving prediction qualitatively. Figure 1 shows two examples where conspicuous incorrect predictions at the N-terminus and the C-terminus are removed.

### Adding gap parameters to the model improves contact predictions overall

**Figure 2:**
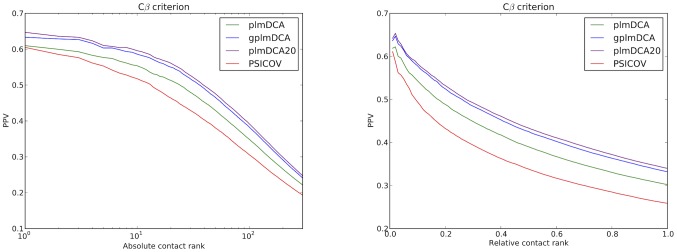
Prediction precision (PPV), average over all proteins in the main test data set. The curves show for PSICOV, plmDCA, gplmDCA and plmDCA20 the average of the number of correct predictions in the *n* highest scoring pairs divided by *n*. Left panel: PPV for absolute contact index; the horizontal axis shows *n*. gplmDCA and plmDCA20 yield higher absolute PPV than plmDCA for all *n*. PSICOV is more often right than plmDCA in its prediction of the few first (strongest) contacts (*n* = 1), but is inferior to both plmDCA20 and gplmDCA for this test set. Right panel: PPV for relative contact index (fraction of protein length). the horizontal axis shows (*n*/*N*).

Using a large test set, the *main data set* as described in Methods, we have found that adding gap parameters increases positive predictive value (PPV) for a large majority of all proteins in the data set. This increase holds for our main criterion (*Cβ* criterion) for both absolute PPV and PPV relative to protein length, see Figure 2. The average relative improvement of gplmDCA over plmDCA, as measured by mean absolute PPV, is 10.4% (8.6% to 12.2% within a 95% confidence interval). In this paper our focus is on the possibility of learning models which lead to better contact prediction, and not of learning a given model more or less well.

To set a scale of the improvement we include however in the comparisons in Figures 2 and 4 also PSICOV [20], another leading approach to the DCA, which can be understood to learn the same model as plmDCA, but by a different inference method.

Supporting Information S1 contains results of the analysis conducted in this paper based on our former criterion (8.5 Å heavy atom criterion) for the sake of immediate backwards comparability with previous work [22], [24].

### Adding gap parameters to the model improves individual contact predictions

**Figure 3:**
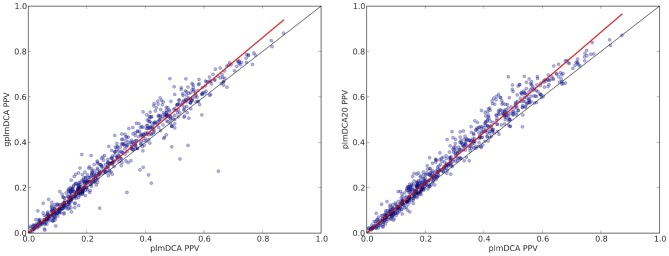
Contact prediction accuracy (mean absolute PPV) for proteins in the main test set by plmDCA (abscissa) vs gplmDCA (ordinate) in left plot and plmDCA vs plmDCA20 in the right plot. Most of the points fall above the diagonal indicating that gplmDCA is more accurate than plmDCA for most of proteins in the test set. Data points can be fitted a straight line by Ordinary Least Squares regression, with slope 1.0764±0.005 (*R* ^2^ = 0.987) indicating that gplmDCA is generally relatively more accurate than plmDCA the more accurate is plmDCA itself. The slope of OLS regression line for plmDCA20 is 1.106±0.004 (*R* ^2^ = 0.992).

A regression analysis of prediction accuracy, as measured by absolute PPV, reveals clear systematic differences between plmDCA and gplmDCA. As shown in Figure 3 the overall advantage of gplmDCA primarily arises from proteins where PPV is relatively high, *i.e.* where prediction by plmDCA itself is accurate.

**Figure 4:**
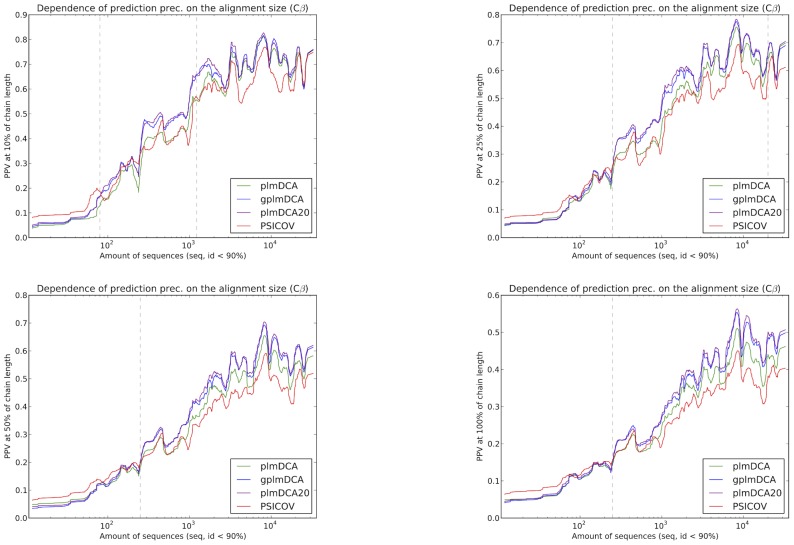
Contact prediction accuracy for proteins in the test set by plmDCA20, gplmDCA and plmDCA *vs* number of homology reduced sequences in the alignment (maximum 90% sequence identity), when considering top 10%, 25% (top row), 50% and 100% (bottom row) contacts, 100% being the same number of contacts as the number of amino acids in the protein. The advantage of gplmDCA and plmDCA20 is particularly interesting in ranges highlighted by vertical dotted lines. For the top 10% and top 25% (top row) these ranges are approximately 60–2500 and 250–23000 sequences, while for the top 50% and top 100% (bottom row) they extend from about 250 sequences in the alignment and upwards. PSICOV outperforms both plmDCA and gplmDCA when there are less than about 100 sequences in the alignment.

Quantitative statistics of this effect are summarized in Table 1. Including all 729 proteins in the main test set we find that in 82% of the cases gplmDCA does at least as well as plmDCA, but if we include only the 608 instances where the PPV from both plmDCA and gplmDCA are larger than a relatively low cut-off of 0.1 this fraction rises to 86%, eventually reaching 91%.

**Table 1:** Numbers and fraction of proteins where gplmDCA performs better than plmDCA.

		gplmDCA		plmDCA20	
Cutoff	Proteins	Better	Better or equal	Better	Better or equal
0.5	128	109 (0.85)	116 (0.91)	117 (0.91)	122 (0.95)
0.4	227	194 (0.85)	205 (0.90)	206 (0.91)	215 (0.95)
0.3	322	277 (0.86)	290 (0.90)	294 (0.91)	304 (0.94)
0.2	441	371 (0.84)	395 (0.90)	400 (0.91)	417 (0.95)
0.1	608	475 (0.78)	524 (0.86)	521 (0.86)	561 (0.92)
ALL	729	522 (0.72)	597 (0.82)	579 (0.79)	639 (0.88)

In each row all proteins in the data set are included for which the PPV from both plmDCA and gplmDCA is larger than the cutoff value given in the first column. The full data set (last row) consists of 729 proteins for 522 (72%) of which gplmDCA performs better than plmDCA. In the most stringent selection (first row) there are 128 proteins where both plmDCA, plmDCA20 and gplmDCA have a PPV of at least 0.5. In this set gplmDCA performs better on 109 (85%) of the instances. By the same criteria, plmDCA20 performs slightly better than gplmDCA, outperforming plmDCA for 579 proteins (79% of all) and performing better in 117 cases (91%) out of 128 proteins highly amenable to contact prediction by these methods.

It is evident that the expected utility of DCA-like contact prediction is heavily dependent on the information content in the input alignment. The information content is closely correlated to the number of unique protein sequences in the alignment. Until recently, it has been a rule of thumb that one needs at least 10 times as many sufficiently diverse proteins in the alignment as there are amino acids in the protein in question. That meant that contact prediction with alignments of fewer than 1000 sequences was considered unfeasible.

### Adding gap parameters to the model leads to improved predictions when there are few sequences

As shown in Figure 4 the improvement in prediction performance by using gplmDCA depends on how many sequences there are in an alignment. When considering the top ranked 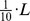 contacts per protein, where *L* is protein length, the improvement is centered in an interesting intermediate range of approximately 90–2500 sequences with at most 90% sequence similarity, while gplmDCA and plmDCA are similar in performance when the number of sequences is less than 90 (where it is poor) or more than 2500 (where it has saturated at a PPV around 65%). Even with as few as 300 unique sequences in alignment, gplmDCA is able to achieve 40% positive prediction rate for these highest ranked contacts. As more contacts are considered, the range where gplmDCA holds an advantage moves successively to proteins with more sequences. A proposed explanation of these observations is that the less information (sequences) are available, the more prominent the confounding factor of the gaps becomes for plmDCA. Introducing gap parameters alleviates this phenomenon, increasing the prediction precision for top ranked contacts for information-poor alignments and improving the amount of correct contacts predicted for the information-rich alignments.

### Discarding the couplings involving gaps in scoring leads to analogous effect as introduction of gap parameter

An alternative method of accounting for gap stretches in the inference is to not include the inferred couplings involving gap variable in the final scoring of coupling matrices *J*. This approach we subsequently denote as plmDCA20. While ignoring gap observations in their entirety, leads to diminished prediction precision [24], discarding the contributions from the gap state in computing the average product corrected Frobenius norm, does indeed improve the prediction precision on a level exceeding the improvements achieved by gplmDCA. The average relative improvement of plmDCA20 over plmDCA, as measured by mean absolute PPV, is 13.1% (11.5% to 14.7% within a 95% confidence interval). On the data set used in this paper, plmDCA20 is notably more precise than gplmDCA, with the relative improvement of 3.9% (95% confidence interval 2.5% to 5.1%). It is important to note, that inferred couplings involving gaps are discarded only *after* gauge fixing, which means that gap observations are included in the inference process and consequently contribute to scoring, although in an indirect way.

## Discussion

While the set of proteins reported in this work is significantly more “difficult” than the proteins reported in recent work on the subject, it is evident that extending the model with a gap term or discounting couplings involving gaps upon scoring, significantly increase the accuracy of prediction. This improvement can be attributed to incremental developments in three aspects, which we call the *three dimensions of contact prediction*: data, model and method. While each of these aspects has been shown to have a non-negligible impact on the accuracy of contact prediction on its own, this work suggests they should not be considered separately, but rather in unison.

### The data

**Figure 5:**
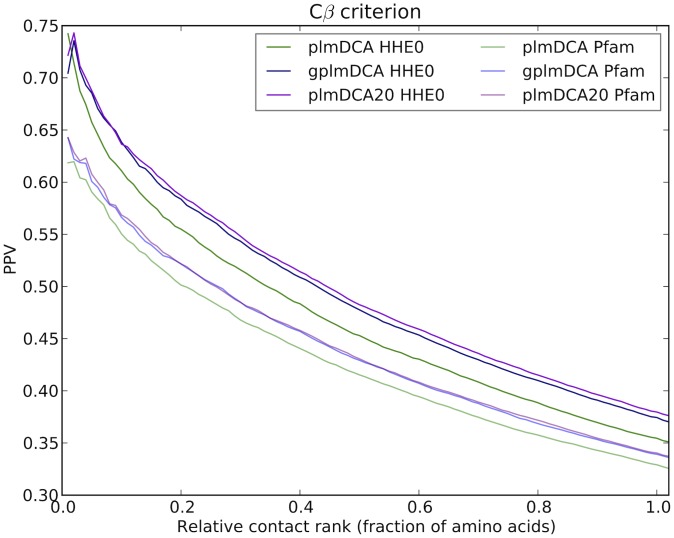
Prediction performance as assessed by relative PPV and *Cβ* criterion for gplmDCA, plmDCA20 and plmDCA run on Pfam and HHblits alignments in the reduced test data set. The reduced test data set comprises the proteins in the main test data set where a comparison can be made to Pfam alignments, as described in Methods.

The extensive benchmark performed for the purposes of the paper has validated our previous claim that proper input alignment matters for accurate contact prediction [30]. To compare HHblits and Pfam alignments we have from our main data set constructed a *reduced data set* of 384 proteins. As shown in Figure 5 and Table 2, gplmDCA and plmDCA20 have a larger advantage over plmDCA on HHblits alignments than on Pfam alignments. Note that plmDCA on HHblits alignments has comparable prediction performance to either gplmDCA or plmDCA20 on Pfam alignments, confirming again the importance of the data dimension in contact prediction.

**Table 2:** Comparison of the effect of different inference methods and alignment sources on precision of contact prediction, based on the reduced data set of 384 proteins.

Method	HHblits			Pfam		
	L/5	L/2	L	L/5	L/2	L
plmDCA	0.54	0.44	0.34	0.51	0.42	0.33
gplmDCA	0.58	0.47	0.37	0.52	0.43	0.34
plmDCA20	0.59	0.48	0.37	0.52	0.43	0.34
decgplmDCA (1)	0.57	0.47	0.36	0.52	0.43	0.34
decgplmDCA (4)	0.56	0.46	0.36	0.52	0.43	0.34
decgplmDCA (9)	0.52	0.43	0.33	0.51	0.43	0.33
PSICOV	0.49	0.38	0.29	0.42	0.33	0.25

L/5, L/2 and L denote the precisions at respective amounts of contacts considered for evaluation, where L is the length of protein. Runs of gplmDCA with decimation are denoted as decgplmDCA (N), where N indicates the amount of decimation rounds. Difference betwwen individual methods is significantly more perceptible when considering HHblits alignments than Pfam alignments. On this set plmDCA and gplmDCA perform comparably, with plmDCA20 showing slightly higher positive predictive value for the top ranked contacts (0.59 vs 0.58).

**Figure 6:**
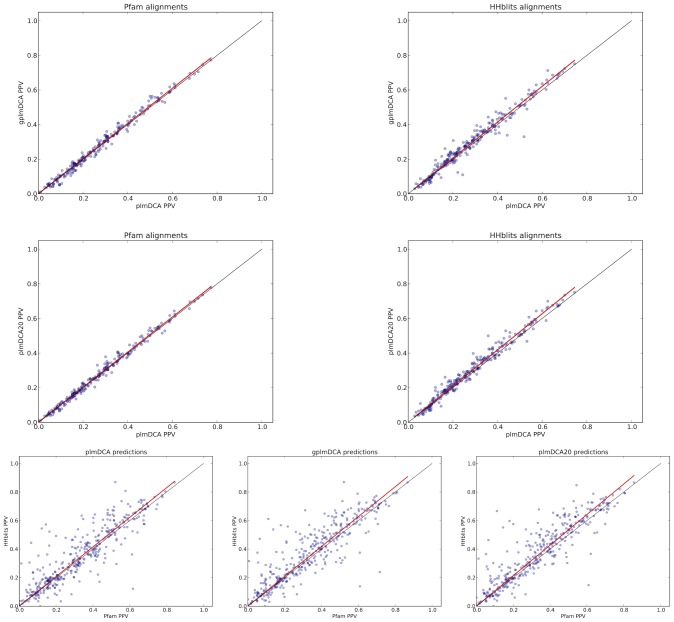
Scatter plots of prediction by absolute PPV and *Cβ* criterion for individual proteins in the reduced test data set. Top row shows, analogously to Figure 3 (in Results, for the main data set), gplmDCA vs plmDCA for Pfam alignments (left panel) and for HHblits alignments (right panel). Center row shows analogous data, but for plmDCA vs plmDCA20 comparison. Bottom row shows prediction for HHblits alignments vs Pfam alignments using plmDCA (left panel), gplmDCA (central panel) and plmDCA20 (right panel).

On the level of single proteins, both with Pfam alignments and HHblits alignments, gplmDCA has a clear advantage over plmDCA in terms of the prediction precision, see top row of Figure 6. The difference is more pronounced for HHblits alignments, which can be quantified by the slope of OLS regression line, that is 1.034±0.005 in case of HHblits alignments, but only 1.023±0.003 for Pfam alignments. In the other dimension of the same test, gplmDCA gains more from use of HHblits over Pfam than plmDCA (bottom row of Figure 6), with the regression line slopes of 1.047±0.13 for gplmDCA and 1.033±0.013 for plmDCA.

For plmDCA20, the same effect is also observable (see middle row of Figure 6), with a comparable slopes of regression lines, that is 1.053±0.004 for HHblits alignments and 1.025±0.003 for Pfam alignments in the dimension of the alignment. In the dimension of the inference method, plmDCA20 benefits from HHblits alignments slightly more than gplmDCA, with a slope of OLS regression line equal to 1.072±0.013 (bottom row of Figure 6).

### The model

Contact prediction in DCA has hitherto been considered in terms of a pairwise interaction model, typically motivated by *maxentropy* arguments *cf* [27]. In a context where one tries to learn from all of the data and not from a reduced set of observables such as *e.g.* pair-wise correlation functions, *maxentropy* arguments do not apply, and there is a vast array of possible models that could describe the biological reality more accurately. We have shown here that the addition of what is arguably the simplest and most obvious non-pairwise term, the gap term, does make a significant difference to the quality of resulting contact predictions, although the beneficial effect is not always consistent and similar improvement may be achieved by correcting the scoring method. Therefore we posit that *the pairwise interaction term is not the end of the story, but rather a prelude*, and that there remains a lot that can still be done in respect to constructing data models that more accurately reflect the evolutionary relationships in proteins.

### Inference

As previously shown by some of us [22], [24], [30], pseudo-likelihood maximization tends to outperform mean-field DCA (mfDCA) [17] and sparse inverse covariance methods (PSICOV) [20] in terms of the prediction precision. Recently, a decimation strategy for improving the inference of the topology of an Ising model has been proposed in the context of pseudo-likelihood inference [32]. The idea is to run the inference several times, setting a fraction of the weakest couplings to zero after each run and constraining them to remain zero in consecutive runs. In order to test whether this additional step improves protein contact prediction, we adapted the method for the asymmetric inference of the Potts Model used in the present work. The implementation details can be found in the Supporting Information S2.

We have benchmarked our implementation of gplmDCA with decimation (decgplmDCA) basing on the reduced test set used for comparison between Pfam and HHblits. According to our results, inference with decimation does not produce on average significantly different results in comparison to inference without decimation, when run on Pfam alignments. For HHblits alignments, decimation-aided inference performs roughly equally well to the regular one, until roughly 50% of couplings are set to 0. From this point on, the average prediction performance starts decreasing, as can be seen in Supporting Information S2.

Since the matrix of coupling strengths resultant from the inference should be sparse, as there are significantly more non-contacting amino acid pairs than contacting ones, decimation is expected to be beneficial in a general case. We believe, that the fact that we observed no such effect indicates that more work is needed on designing the decimation-aided inference method in unison with the data model and data itself.

### More accurate contact maps

The improvement in terms of the average PPV over the whole protein set, as well as the fraction of proteins for which gplmDCA and plmDCA20 produce more accurate predictions, cannot be be underestimated, but is not the only distinguishing feature of these methods. Eliminating strong couplings induced by gaps in the alignments allows for detection of relatively weaker ones, which may be important for the future applications of the method, such as contact-assisted protein folding.

**Figure 7:**
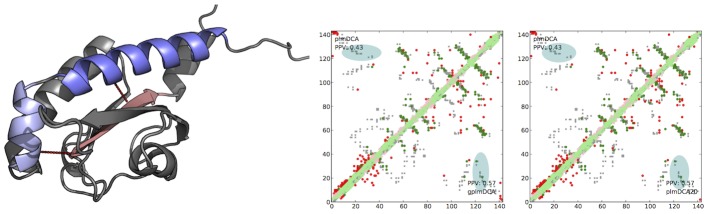
Difference in contact prediction between plmDCA and gplmDCA for sensor domain of histidine kinase DcuS from *E.coli* (pdbid:3BY8:A). Left figure: protein structure, with some of contacts uniquely predicted by gplmDCA marked by dashed lines. Center and right: contact maps, with the region of interest marked in faint blue. Predictions by both plmDCA20 and gplmDCA differ slightly, but maintain the same accuracy and uncover additional contacts, important for protein structure prediction.

One example of such contacts being predicted, shown in Figure 7, is the contacts between N-terminal helices (marked in blue) and the *β*-sheet of the sensor domain of histidine kinase DcuS (deposited in PDB as 3BY8:A). This structure is classified in CATH [33] as a two-layer *α*/*β* sandwich and while plmDCA is able to position strands of the *β*-sheet in a correct order, it fails at predicting contacts between the *α*-helices of the sandwich and the *β*-sheet. As can be seen in central panel Figure 7, gplmDCA in addition to the already predicted contact between residues 34 and 113 (green dot next to the blue region) predicts also contacts between residues 34 and 121, as well as 21 and 126. This in theory should allow for proper positioning of helices in case of structure prediction. For this protein plmDCA20 also predicts these additional contacts and while plmDCA20 predictions are not identical to gplmDCA ones, both methods achieve the same prediction precision.

### Wrong predictions

The addition of a gap term, while beneficial for vast fraction of proteins, occasionally results in lower prediction accuracy in comparison to the inference performed on a model without gap term (plmDCA).

**Figure 8:**
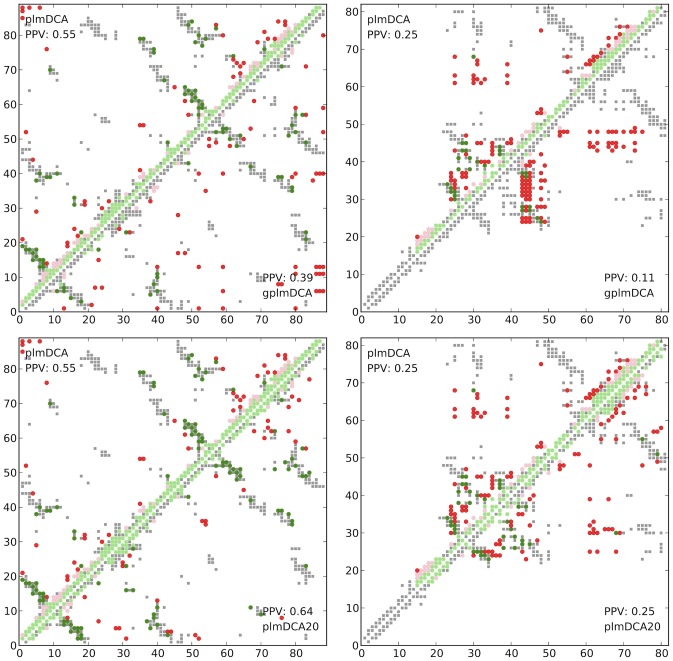
Mispredictions. Among the 729 proteins plotted in Figure 3 there is less than 5% prominent outliers where plmDCA (model with no gap parameters) clearly does better than gplmDCA (model with gap parameters). Upper row depicts gplmDCA predictions, lower — plmDCA20. Left panels show the contact maps of protein S, where gplmDCA wrongly predicts a number of spurious contacts between N- and C- terminii Right panels, contact maps of transcription elongation factor Spt4. The prediction artifacts of gplmDCA are not detectable in plmDCA20 predictions. For further discussion, see main text.

One of the most striking examples (see Figure 8) is protein S, a member of the beta gamma-crystallin superfamily, from *Myxococcus xanthus* (deposited in PDB as 1NPS:A), which is one of the most prominent outliers in Figure 3. For this protein plmDCA predicts contacts allowing theoretically for proper assembly of protein, with most of the false positives concentrating in the areas immediately close to diagonal (with sequence separation ≤10). On the the hand gplmDCA predicts here significantly fewer such false contacts, but at the same time neglects to predict nearly all close range contacts. Another example depicted in panel (B) of the same figure is transcription elongation factor Spt4 from *Pyroccocus furiosus* (deposited in PDB as 3P8B:A). In this case, all the contacts predicted by gplmDCA concentrate in rectangular regions between residues 24–49, 53–56, 59–75, which we believe could be due to the high percentage of sequences with identical gap distribution in the alignment, either (case 1) 1–23, 50–52, 56–59, 77–81 (31.7% of sequences) or (case 2) 1–23, 50–52, 56–59, 64–65, 74–81 (28.4% of sequences).

We believe that the sub-par prediction accuracy for these and most of the other outliers is due to the way input multiple sequence alignment has been constructed. HHblits (the method used for constructing input multiple sequence alignments) tends to result in multiple sequences in the alignment containing identical distributions of gaps, which causes gplmDCA to assign lower coupling strengths to the gap-rich regions. Alignments of similar size produced by different methods (i.e. jackhmmer, data not shown), do not seem to exhibit such a behavior. Despite this shortcoming, we have found that HHblits alignments are highly suitable for contact inference (*cf.* the data section).

In contrast to gplmDCA, we did not find any proteins for which plmDCA20 performs significantly inferior to the original plmDCA (as demonstrated by Figure 3). In particular, for proteins discussed above plmDCA20 provides predictions on par or better than plmDCA. With an exception of approximately 5% proteins, prediction performance of plmDCA20 and gplmDCA is comparable for our test set.

### Folding

Elimination of artifacts in predicted contact maps, as well as increased sensitivity (predicting correct contacts between more secondary elements) in comparison to plmDCA, coupled with increased prediction precision, strongly suggest that gplmDCA and plmDCA20 should provide valuable input for the future *ab-initio* protein structure prediction attempts. The previous incarnation of pseudo-likelihood maximization for direct coupling analysis (plmDCA) has been successfully used for protein structure prediction endeavors (c.f. [12]) as it objectively provides higher prediction accuracy than other methods (as demonstrated, for example in [30]). As both methods presented in this paper are at the same time faster and more accurate than the version used in reported structure prediction work, we strongly recommend them for future use.

## Conclusions

Contact prediction has advanced greatly in the last five years, reaching a level of accuracy which was previously believed to be unattainable. We have shown here that the three *dimensions* of data, model and method are all important for overall prediction success, and we have shown that one can can significantly improve prediction along the second dimension by going beyond pairwise *maxentropy* models mainly used in the field up to now. Finally, we have shown that the gap correction behavior can be achieved by alternative method of scoring the resultant coupling matrices. We believe that these are only the first steps in a rational approach to incrementally improve contact prediction, and that with the ongoing explosion in the number of available protein sequences much further progress should be possible on these issues.

## Methods

The Direct Contact Analysis (DCA) as introduced in [34] and [16] is a family of methods to predict contact between amino acid pairs from a multiple sequence alignment (MSA) [17], [18], [20]–[22], [26], [27], [30], [35]–[38]. Learning predictive models of amino acid contacts depends on which sequences are used to build the alignment and by which methods they are aligned (*Input data*), which model one tries to learn from the data (*Model*) and how a model is learned from the data (*Inference method*). We describe below our approach along these three dimensions in turn. The perceived quality of prediction then depends on how the model is used and how it is benchmarked, as we describe below (Prediction and benchmarking metrics).

### Input data

In a substantial fraction of the contributions to the development of DCA contact predictions have been based on MSAs obtained from the Pfam protein families database: [3], [39]. However, as recently shown by one of us in [30], and as also shown here (see Discussion), these alignments are not the optimal input for DCA and DCA-like methods.

Instead of PfamA alignments, we use a state-of-art homology detection method HHblits [40], based on iterative comparison of Hidden Markov models (HMMs). This approach is able to arrive at very accurate multiple sequence alignments, tailored to the protein of interest, while still including remotely homologous proteins.

We have constructed a heterogeneous set of 729 protein chains of known structure, sampled from Protein Data Bank which we refer to as *main test set*. This set is an amalgam of four smaller data sets as follows:

• 150 proteins reported in PSICOV paper [20].

• ∼120 proteins with known structures, with relatively few detectable homologous proteins of known sequence.

• ∼180 proteins of the most common Structural Classification of Proteins (SCOP) folds [41].

• ∼280 proteins sampled at random from PDB.

We excluded from the main test set proteins that were significantly too long for a reasonable contact prediction (the mean and median lengths of a protein in the considered set are 168.4 and 150 amino acids correspondingly, with maximum of 494 amino acids), or not compact enough (not having enough long-range contacts), probably stabilized by interaction with their environment. We did not exclude multimeric proteins, or filter out multidomain proteins, though.

The alignments in the main test set have been constructed using HHblits, as contained in HHsuite 2.0.16 with a bundled uniprot20_2013_03 database. We have run five iterations of search, with a E-value cutoff of 1, allowing for inclusion of distantly homologous protein in the alignment. The search was conducted without filtering the result MSA (-all parameter), without limiting the amount of sequences allowed to pass the second prefilter and allowing for realigning all the hits, hence obtaining the most information-rich and accurate alignment at cost of increased running time.

To compare Pfam and HHblits-based predictions we have from the main test set also constructed a *reduced test set* by the following procedure. For each of the proteins in the main test set we searched for its PDB identifier against an official Pfam-PDB mapping, to identify the longest Pfam family corresponding to this protein (in case of potential multiple Pfam hits per PDB identifier). This resulted in alignments for 481 proteins, reflecting *inter alia* the fact that not all proteins in the main test set have an official Pfam-PDB mapping. Then we identified the sequence in the appropriate Pfam alignment which is closest to the sequence of protein in question by Smith-Waterman algorithm using BLOSUM100 matrix. From this set we reject alignments where we the number of residues in both sequences aligned to gaps is more than 50% of length shorter of sequences plus length difference between sequences, and subsequently we trim the Pfam alignment to only the columns aligned to protein in question. Finally, the reduced test set contains 384 proteins with both Pfam and HHblits MSAs which form the input for plmDCA, plmDCA20 and gplmDCA in the comparisons presented in Discussion and Figures 5 and 6. The comparison is there done by filtering down the predictions to include only the columns present in the Pfam alignments.

Protein sequences present in sequence database (and hence used for alignments in this work) are biased towards sequences from genomes of organisms that are of special interest to humans. Many such sequences are closely similar, and following [16] sequences that are more similar than some threshold are reweighted before being used in a DCA. We here use the reweighting recently described in [24], with threshold 0.1, that is, by reweighting sequences that are more than 90% identical.

### Model

A multiple sequence alignment can be considered as samples from an unknown probability distribution. Each row, corresponding to one protein in the alignment, is then one of the *q^N^* possible realizations of a random variable which at each of the *N* positions along the row can take *q* = 21 different values (the amino acid or the gap symbol at that position). The (unknown) probability distribution is, in principle, the result of the complete evolutionary history of all forms of life, and is therefore a very complicated object. However, it is not necessary to know the probability distribution exactly to extract useful information.

The Direct-Coupling Analysis (DCA), as introduced in [34] and [16], assumes that the probability distribution is the *Potts Model* of statistical physics [42]: 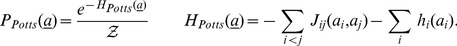
(1)


The use of the Potts model in the DCA has often been motivated by *maxentropy* arguments *cf* [27]. As we base our approach an inference method which uses all the data (see below), we cannot refer to *maxentropy* principles. Instead, one may observe that it has been found in many branches of science and engineering, that probability distributions over a collection of a large number of similar objects often obey a large deviation principle [43]. The full distribution *P* can then be written as *P*(*a*)≈exp(−*L*(*a*)), where the function *L* in the exponent is “simple”, a classical example being the Gibbs-Boltzmann distribution of equilibrium statistical mechanics. An unknown probability distribution can then be expanded in a series 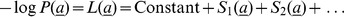
(2)where the first order contribution *S*_1_ (linear) contains terms only depending on one component of *a*, the second order contribution *S*_2_ (bi-linear) contains terms depending on two components of *a*, and so on. If *L* in fact *is* simple, then a low order truncation should give a useful approximation to *P*, and the Potts model of (1) is nothing but the truncation of (2) after the second order terms. We note that hierarchies of exponential probability distributions have non-obvious properties, and may for instance be taken as a basis of an invariant decomposition of the entropy [44].

Any multiple sequence alignment procedure typically produces stretches of gaps, a fact which is obvious by visual inspection. It is therefore an immediate observation that a real MSA data cannot be a set of independent realizations of the rather simple model in (1), since such stretches of one and the same variable (the gap variable) are very unlikely to occur in a random variable drawn from the distribution (1). In a DCA based on (1) we manifestly learn from data a model which does not generate the same data. We therefore hypothesized that by learning a model which describes the data better, we might also better predict amino acid contacts.

To investigate this we introduced additional gap parameters and try to learn 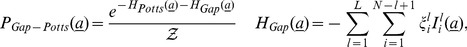
(3)where the 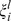 are new parameters describing the propensity of a site *i* to be the beginning of a gap of length *l*, 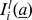 is an indicator function which takes the value 1 if there is a gap of length *l* beginning at site *i*, and otherwise zero, and *L* is a meta-parameter, the largest gap length included in the gap parameters. We set *L* to the largest gap length found in a given alignment. The number of additional parameters to be learned is thus not larger than *NL*, to be compared to the number of parameters already used in (1), which is about 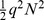.

### Inference method

The benchmark of learning a model from data is maximum likelihood where one chooses the probability distribution in a class which minimizes a negative-log-likelihood function *L*. The main problem in learning (1) from data by maximum likelihood is that the normalizing constant (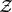) cannot be evaluated exactly and efficiently in large systems, and that therefore maximum likelihood learning can only be done approximately *e.g.* by variational methods [45]. Therefore, we instead use the weaker learning criterion of pseudo-likelihood maximization [46], first applied in the DCA setting by one of us in [22]. A further issue is that the number of parameters in a Potts model based DCA is (typically) larger than the number of observations (number of sequences in an MSA), and regularization is therefore necessary. We here base our work on the recently developed *asymmetric pseudo-likelihood maximization* [24], which is considerably faster than the version presented in [22] while showing essential identical performance as a predictor of amino acid contacts.

Learning the new model including (3) is especially convenient using the pseudo-likelihood maximization approach. We have developed a new code gplmDCA based on the asymmetric version of plmDCA of [24].

### Prediction and benchmarking metrics

The outcome of learning a model of the Potts type is a set of pairwise interaction coefficients *J_ij_* (*a_i_*, *a_j_*). For each pair (*i*, *j*) (each pair of positions) this is a matrix in two other variables (*a_i_* and *a_j_*) and how an inferred interaction is scored depends on which matrix norm one uses. We here use the Frobenius norm augmented by the Average Product Correction (APC), as introduced in the context of DCA by one of us in [22], and order the pairs (*i*, *j*), for each multiple sequence alignment, by the value of this score.

An alternative method of handling the gaps in the alignment (plmDCA20) is to change the scoring function, such that the Frobenius norm is computed only on the 20×20 sub-matrix which does not involve the gap variables. The procedure is to ignore the gap couplings *after* computing the coupling matrix *J*, which is manifestly not the same as ignoring data on the gap variables altogether. Since *L*_2_ penalty in plmDCA enforces the Ising gauge for the couplings, the gap observations *are* used in the inference and consequently contribute to the result, although in a non-trivial way. In our experience (Aurell & Hartonen, unpublished results), ignoring the data on gap variables in the inference does not result in any improvement in the prediction precision.

To benchmark the predictions of the DCA one compares against known crystal structures. In this work we use as the main benchmark criterion, that two amino acids are in contact, if their *Cβ* atoms are at most 8 Å apart in the crystal structure. This we denote as *Cβ criterion* and use predominantly throughout this article. In order to facilitate comparison to previously published work on the DCA we present also an alternate metric that considers the amino acids to be in contact if any of their heavy (non-hydrogen) atoms are at most 8.5 Å apart. This metric is denoted as 8.5 Å *heavy atom criterion* We strongly believe that this metric tends to label unduly high fraction of short-range contacts (i.e. contact separated by less than 8 positions in sequence space) as positive. At the same time original plmDCA predicts significantly more short-range contacts in comparison to the background distribution in native protein structures. Both observations in conjunction cause the improvements to the prediction precision to be less perceptible. We demonstrate this effect in Supporting Information S1.

In this article we use the terms *precision* and *PPV* (positive predictive value) interchangeably, with metric denoting the ratio of true positives to all predictions (within a certain count threshold). In line with previously published work on contact prediction, we consider only the contacts with sequence separation greater or equal to 5 amino acids (we do not consider very short range contacts, that is contacts between amino acids *i* and *j* when 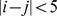).

By the term *weighted moving average* with window *w*, authors understand a weighted arithmetic mean of a value at a given position and *w* values on either side of the center position, thus resulting in 2*w*+1 values to be averaged. The central position is scaled with weight *w*, whereas the weights decrease in arithmetic progression while moving away from the center (i.e positions −1 and +1 are scaled with weight *w*−1, whereas positions −2 and 2 with weight *w*−2 etc.).

### Availability

The code of gplmDCA is freely available at http://gplmdca.aurell.org. This website contains also a link to all the data the benchmark is based on, that is: multiple sequence alignments, predicted couplings (both plmDCA and gplmDCA), protein structures and contacts derived from them.

## Supporting Information

Supporting Information S1 Metrics of contact prediction correctness and results with heavy atom distance threshold of 8.5 Å.
(PDF)

Supporting Information S2 Decimation. Implementation details and effect on prediction precision.
(PDF)
