# Supplementary material for: Improving Contact Prediction along Three Dimensions
Source: PLoS Comput Biol. 2014 Oct 9;10(10):e1003847. doi: 10.1371/journal.pcbi.1003847 (PMC4191875; doi:10.1371/journal.pcbi.1003847)
Supplement: Supporting Information S1 — Metrics of contact prediction correctness and results with heavy atom distance threshold of 8.5 Å. (PDF) [file pcbi.1003847.s001.pdf]

Supporting Information S1: Metrics of contact correctness

Results using the 8.5Å heavy atom criterion

In this work we have used the *Cβ criterion*, that a pair of amino acids are in contact in a crystal structure if their Cβ atoms (Cα in case of Glycines) are not more than $\leq$ 8Å apart. This kind of contact evaluation criterion has been used for some time in the biannual Critical Assessment of protein Structure Prediction (CASP) competition, and is considered standard in the field of protein structure prediction [5, 6]. In the context of DCA it was used, with a threshold of 8Å, in [3] and [8].

No fixed criterion of this kind will be perfect. The threshold to use depends on the desired trade-off between false negatives and false positives, and that depends on the intended application. For a reasonable threshold, such as around 8Å, there will be some pairs of amino acids which satisfy the Cβ criterion, but nevertheless probably do not make any contact, at least in one given crystal structure, and there will be some pairs of amino acids which do not satisfy the Cβ criterion, but where the side chains in fact do make contact. In both cases the residue types will matter, an aspect which is not taken into account by the Cβ criterion.

Several earlier publications on DCA have, following [7], used an alternative criterion which we call the *heavy atom criterion*, where a a pair of amino acids are taken to be in contact if the distance between the two closest heavy (i.e. non-hydrogen) atoms of the two amino acids in question is less than some threshold around 8Å. To facilitate comparison we show in this Supporting Information results using the heavy atom criterion with a threshold of 8.5Å, as used by one of us in [1].

Figures and tables in this Supporting Information are numbered identically as in the main paper, and are based on the same data, the only difference being that we use throughout the 8.5Å heavy atom criterion. It is evident that the 8Å Cβ criterion is more stringent than the 8.5Å (or 8Å) heavy atom criterion. A main difference will therefore be that nominal PPVs will be higher using the heavy atom criterion, other differences will be pointed out in figure captions. We note that a heavy atom criterion with substantially smaller thresholds have also been proposed in the literature, such as 6Å in [3], 5Å in [4] and 4.5Å in a paper from before the DCA era [2]. We do not here make any comparisons to

the heavy atom criterion with these choices of thresholds.

This work demonstrated that plmDCA is vulnerable to overprediction of short-range contacts, due to the way it handles gap stretches in the input multiple sequence alignments. On the other hand threshold of 8.5 Å for heavy atom distances tends to assign true contact label to vast majority of amino acids separated by 8 positions or less in the sequences space. These two in tandem cause in our opinion plmDCA to attain unduly high prediction precisions, when measured using this metric. In order to demonstrate it, we have plotted the measured prediction precision using variable thresholds for Cβ and heavy atom criteria in Figure 7. In particular, we would like to call attention to the fact, that an entirely random prediction, with identical distribution of contact separations as plmDCA (that we denote as RandomPLM in the plots) achieves over 50% higher prediction precision than the random prediction with distribution of contact separations observed in real structures (denoted as Random). We believe that lack of perceptible difference between plmDCA and gplmDCA or plmDCA20 can be at least partially attributed to this phenomenon, as further demonstrated by Figure 8.

References cited in this Supporting Information are listed in a separate bibliography.

		gplmDCA		plmDCA20	
Cutoff	Proteins	Better	Better or equal	Better	Better or equal
0.80	109	86 (0.79)	98 (0.90)	100 (0.92)	106 (0.97)
0.70	200	143 (0.71)	167 (0.83)	171 (0.85)	182 (0.92)
0.60	301	197 (0.65)	230 (0.76)	232 (0.77)	251 (0.83)
0.50	392	243 (0.62)	277 (0.71)	286 (0.73)	310 (0.79)
0.40	494	287 (0.58)	327 (0.66)	342 (0.69)	369 (0.75)
0.30	597	321 (0.54)	368 (0.62)	380 (0.64)	419 (0.70)
0.20	672	348 (0.52)	400 (0.60)	409 (0.61)	451 (0.67)
0.10	722	357 (0.49)	409 (0.57)	424 (0.59)	470 (0.65)
ALL	729	358 (0.49)	412 (0.57)	428 (0.59)	475 (0.65)

Table 1: Numbers and fraction of proteins where gplmDCA performs better than plmDCA. In each row all proteins in the data set are included for which the PPV from both plmDCA and gplmDCA is larger than the cutoff value given in the first column. The full data set (last row) consists of 729 proteins for 358 (49%) of which gplmDCA performs better than plmDCA. In the most stringent selection (first row) there are 109 proteins where all plmDCA, plmDCA20 and gplmDCA have a PPV at least 0.8. In this set gplmDCA performs better on 86 (79%) of the instances. By this metric plmDCA20 shows greater improvement that gplmDCA, both for most accurately predicted proteins and for the whole set.

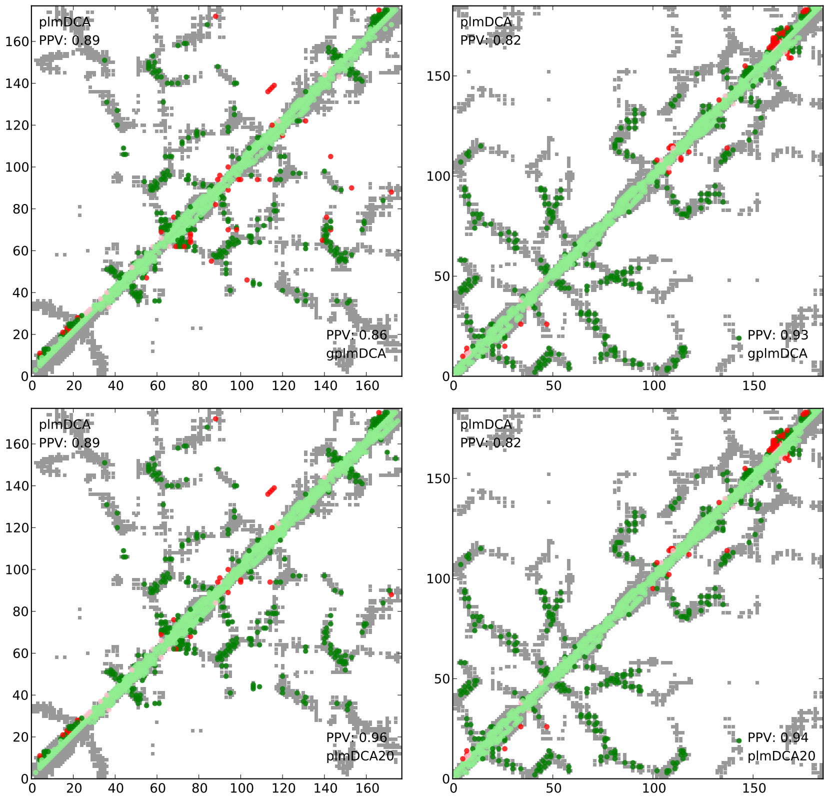

Figure 1: Examples of qualitative contact prediction improvement. Gray squares: contacts observed in crystal structure, Ovals: predicted contacts (green: correctly predicted, red: incorrectly predicted). Predicted short-range contacts (not considered in the assessment) are drawn in pale colors.Top row: comparison of plmDCA and gplmDCA, bottom row: plmDCA and plmDCA20. Left panels: contact prediction maps built by plmDCA and gplmDCA/plmDCA20 using protein sequences homologous to 1JFU:A as explained in Methods. plmDCA here predicts a number of strong couplings at both the N-terminus and the C-terminus which arise from the high sequence variability at both ends of proteins homologous to 1JFU:A, and the many gaps in the multiple sequence alignments at these positions. In gplmDCA these gaps lead to adjustment of gap parameters and not to contact predictions, in plmDCA20 these couplings are not considered in contact scoring. Right panels: analogous results using protein sequences homologous to 1ATZ where gplmDCA and plmDCA20 remove strong spurious couplings at the C-terminus. **Remarks pertaining to the 8.5Å heavy atom criterion:** The PPVs are substantially higher, in the range $0.89-0.95$, and the relative improvement is less pronounced than using the Cβ criterion.

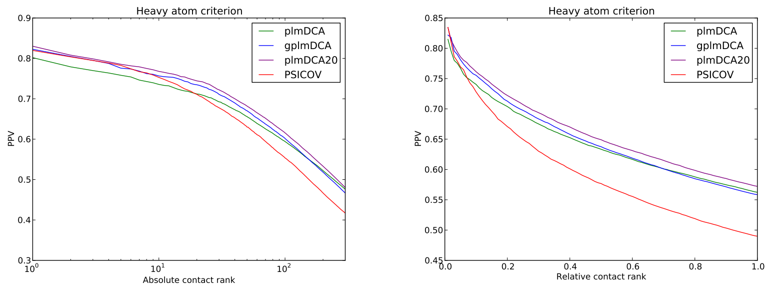

Figure 2: Prediction precision (PPV), average over all proteins in the main test data set. The curves show for PSICOV, plmDCA, gplmDCA and plmDCA20 the average of the number of correct predictions in the n highest scoring pairs divided by n. Left panel: PPV for absolute contact index; the horizontal axis shows n. gplmDCA and plmDCA20 yield higher absolute PPV than plmDCA for all n. Right panel: PPV for relative contact index (fraction of protein length). the horizontal axis shows (n/N). **Remarks pertaining to the 8.5Å heavy atom criterion:** Left panel: plmDCA performs on par with gplmDCA at values of n greater than about 100. Right panel: in difference to the data shown in right panel of Figure 2 in main paper, plmDCA here performs on par with gplmDCA over the full range of n.

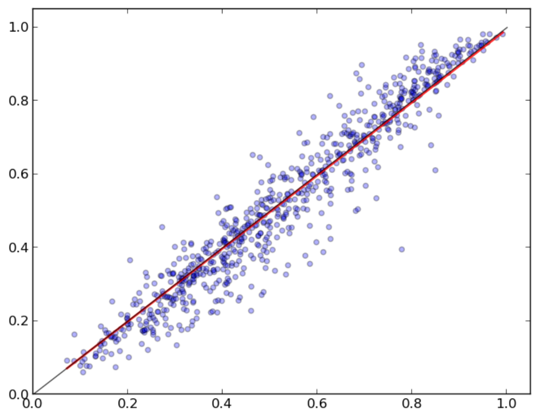

Figure 3: Contact prediction accuracy (mean absolute PPV) for proteins in the main test set by plmDCA (abscissa) and gplmDCA (ordinate). Data points can be fitted by a straight line with slope 0.996 ± 0.004 ($R^2 = 0.986$). **Remarks pertaining to the 8.5Å heavy atom criterion:** By the 8.5 Å heavy atom criterion there is no difference between plmDCA and gplmDCA.

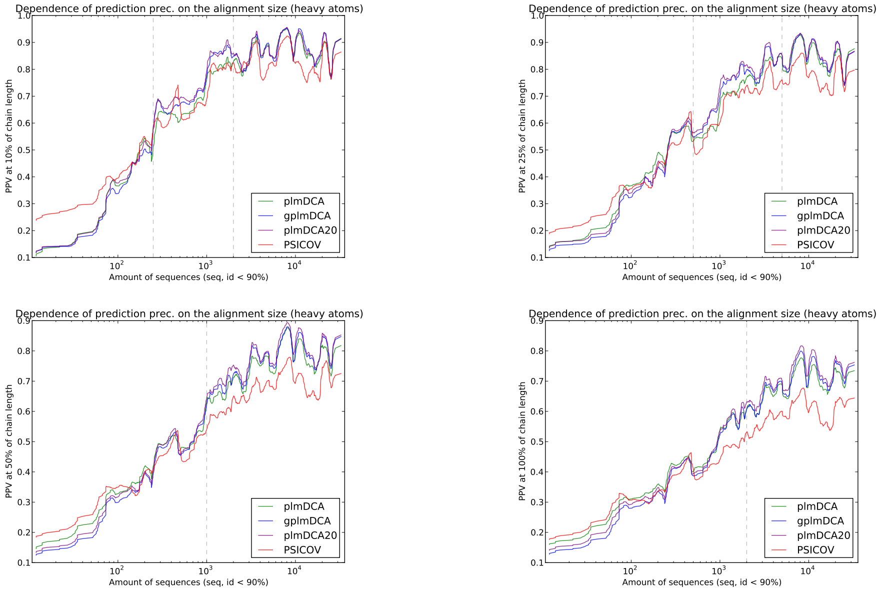

Figure 4: Contact prediction accuracy for proteins in the test set by plmDCA20, gplmDCA and plmDCA *vs* number of homology reduced sequences in the alignment (maximum 90% sequence identity), when considering top 10%, 25% (top row), 50% and 100% (bottom row) contacts, 100% being the same number of contacts as the number of amino acids in the protein. The advantage of gplmDCA and plmDCA20 is particularly interesting in ranges highlighted by vertical dotted lines. For the top 10% and top 25% (top row) these ranges are approximately 250-2000 and 500-500 sequences, while for the top 50% and top 100% (bottom row) they extend from about 1000 sequences in the alignment and upwards. PSICOV outperforms both plmDCA and gplmDCA when there are less than about 100 sequences in the alignment.

Remarks pertaining to the 8.5Å heavy atom criterion: Compared to Figure 4 in the main paper gplmDCA here shows an advantage in more limited ranges.

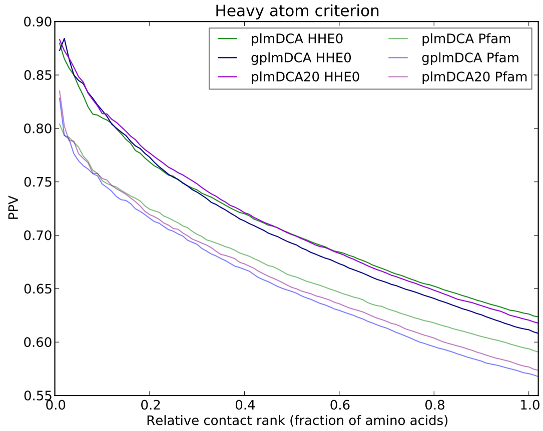

Figure 5: Prediction as assessed by relative PPV and 8.5$\mathring{A}$ heavy atom criterion for gplmDCA, plmDCA20 and plmDCA run on Pfam and HHblits alignments in the reduced test data set. The reduced test data set comprises the proteins in the main test data set where a comparison can be made to Pfam alignments, as described in Methods. **Remarks pertaining to the 8.5Å heavy atom criterion:** As in the data shown in Figure 5 in the main paper, contact prediction is more effective using HHblits alignments. In contrast to Figure 5 in the main paper, neither gplmDCA nor plmDCA20 show any considerable advantage over plmDCA (but c.f. Figure 8)

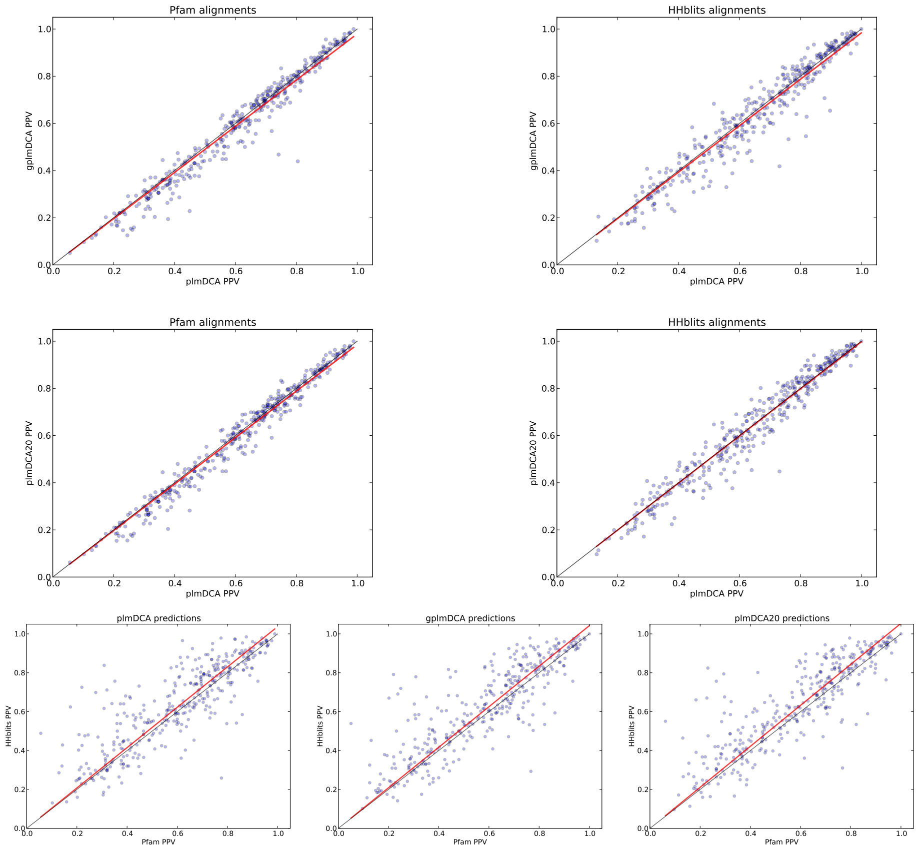

Figure 6: Scatter plots of prediction by absolute PPV and 8.5$\mathring{A}$ heavy atom criterion for individual proteins in the reduced test data set. Top row shows, analogously to Figure 3 (in Results, for the main data set), gplmDCA vs plmDCA for Pfam alignments (left panel) and for HHblits alignments (right panel). Center row shows analogous data, but for plmDCA vs plmDCA20 comparison. Bottom row shows prediction for HHblits alignments vs Pfam alignments using plmDCA (left panel), gplmDCA (central panel) and plmDCA20 (right panel). **Remarks pertaining to the 8.5Å heavy atom criterion:** As in Figure 3 there is no perceptible advantage of gplmDCA or plmDCA20 over plmDCA here.

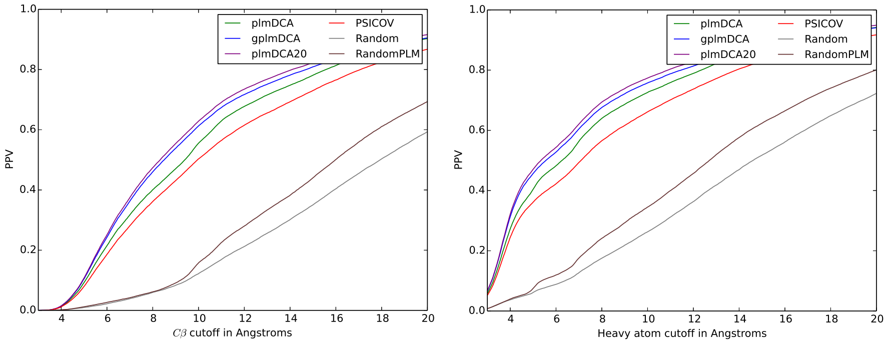

Figure 7: Prediction precision for Cβ (left) and heavy atom metrics (right panel) at L contacts per protein, depending on the chosen distance cutoff. In addition to the methods discussed in the paper, there are two additional lines in the plot: Random and RandomPLM. Random denotes simulated random prediction obtained by permuting the real observed contact matrix, while maintaining the same distribution of contact separations. RandomPLM denotes simulated random prediction obtained by permuting plmDCA predictions. It can be seen that for higher (more permissive) cutoffs, RandomPLM is assigned up to 50% higher prediction precision, which we believe is one of the reasons behind surprisingly good performance of plmDCA in comparison to gplmDCA/plmDCA using 8.5 Å heavy atom metric.

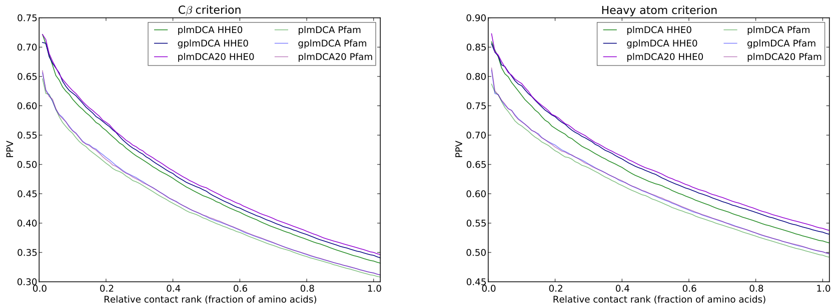

Figure 8: Prediction as assessed by relative PPV and respectively 8 Å Cβ criterion (left plot) and 8.5 Å heavy atom criterion (right plot) for gplmDCA, plmDCA20 and plmDCA run on Pfam and HHblits alignments in the reduced test data set, *considering only contacts separated by at least 10 positions in sequence space*. The reduced test data set comprises the proteins in the main test data set where a comparison can be made to Pfam alignments, as described in Methods. Note, that mutual ordering of methods performance-wise is identical in both plots, on contrary to plot in Figure 5, strongly suggesting that good performance of plmDCA when measured by 8.5 Å heavy atom criterion is due to high fraction of highly local contact predicted, that are ascribed unduly positive contact status.

References

[1] Magnus Ekeberg, Cecilia Lövkvist, Yueheng Lan, Martin Weigt, and Erik Aurell. Improved contact prediction in proteins: Using pseudolikelihoods to infer potts models. *Physical Review E*, 87(1):012707, 2013.

[2] P Fariselli and R Casadio. A neural network based predictor of residue contacts in proteins. *Protein Eng.*, 12(1):15–21, 1999.

[3] David T. Jones, Daniel W. A. Buchan, Domenico Cozzetto, and Massimiliano Pontil. PSICOV: precise structural contact prediction using sparse inverse covariance estimation on large multiple sequence alignments. *Bioinformatics*, 28:184, 2012.

[4] Debora S. Marks, Lucy J. Colwell, Robert Sheridan, Thomas A. Hopf, Andrea Pagnani, Riccardo Zecchina, and Chris Sander. Protein 3D structure computed from evolutionary sequence variation. *PLoS ONE*, 6(12):e28766, December 2011.

[5] Bohdan Monastyrskyy, Daniel D'Andrea, Krzysztof Fidelis, Anna Tramontano, and Andriy Kryshtafovych. Evaluation of residueresidue contact prediction in CASP10. *Proteins: Structure, Function, and Bioinformatics*, 82:138–153, 2014.

[6] Bohdan Monastyrskyy, Krzysztof Fidelis, Anna Tramontano, and Andriy Kryshtafovych. Evaluation of residueresidue contact prediction in CASP9. *Proteins: Structure, Function, and Bioinformatics*, 79:119–125, 2011.

[7] Faruck Morcos, Andrea Pagnani, Bryan Lunt, Arianna Bertolino, Debora S. Marks, Chris Sander, Riccardo Zecchina, Jos N. Onuchic, Terence Hwa, and Martin Weigt. Direct-coupling analysis of residue coevolution captures native contacts across many protein families. *Proceedings of the National Academy of Sciences*, 108(49):E1293E1301, 2011.
