# Supplementary material for: Improving Contact Prediction along Three Dimensions
Source: PLoS Comput Biol. 2014 Oct 9;10(10):e1003847. doi: 10.1371/journal.pcbi.1003847 (PMC4191875; doi:10.1371/journal.pcbi.1003847)
Supplement: Supporting Information S2 — Decimation. Implementation details and effect on prediction precision. (PDF) [file pcbi.1003847.s002.pdf]

Supplementary material 2: Decimation

We wanted to test whether the decimation procedure recently described in [1] for the Ising model can improve the method of protein contact inference presented in this paper. We adapted it therefore for the asymmetric pseudo-likelihood inference of the Potts-model used in the present work and described in [2]. The idea of the decimation is to run the inference algorithm N times and identify after each run n the fraction $n \cdot x$ of all couplings with the smallest absolute values, where x is some constant between 0 and $1/N$. In the consecutive runs these couplings are fixed to 0 and when the algorithm stops a fraction $N \cdot x$ of the couplings has been set to 0 in this manner. Note that in [1] a more sophisticated method of stopping the algorithm is used than to predefine the number of runs. As a first step we were however only interested in seeing whether the decimation has any positive effect. We therefore ran the algorithm several times and assessed the performance in terms of protein contact prediction after every run. Finding the optimal stopping point would then be a second step. The only new parameter was therefore x. We chose to be 0.1, which corresponds to setting to zero 10% of the couplings after every run.

There are several ways to generalize decimation to the asymmetric version of pseudolikelihood-inference: We denote by $\mathcal{L}_i \equiv \mathcal{L}_i(J_i, h_i)$ the pseudolikelihood-function connected to site i dependent on the data and on all couplings and fields acting on site i. We note that the authors in [1] minimize an objective function of the form

$$-\sum_i \log \mathcal{L}_i \tag{1}$$

analogous to one implemented in an earlier version of plmDCA described in [3].

In [2] and in the present paper on the other hand, the functions $-\log \mathcal{L}_i$ are minimized independently for every i. Given that the couplings connected to sites i and j appear in $\mathcal{L}_i$ and $\mathcal{L}_j$, it must be decided what happens if a coupling gets decimated in $\mathcal{L}_i$ but not in $\mathcal{L}_j$ after a certain run. We decided to implement a completely asymmetric version of the decimation. This means that the decimation procedure is used independently for all $\mathcal{L}_i$. This has the advantage that the minimization of the $\mathcal{L}_i$ is still completely asymmetric. The disadvantage is that it might happen that only one of the two estimates obtained for one coupling is decimated. Because the final estimate for the coupling is obtained by taking the mean of the two estimates, the final coupling matrix is not necessarily sparse after the decimation.

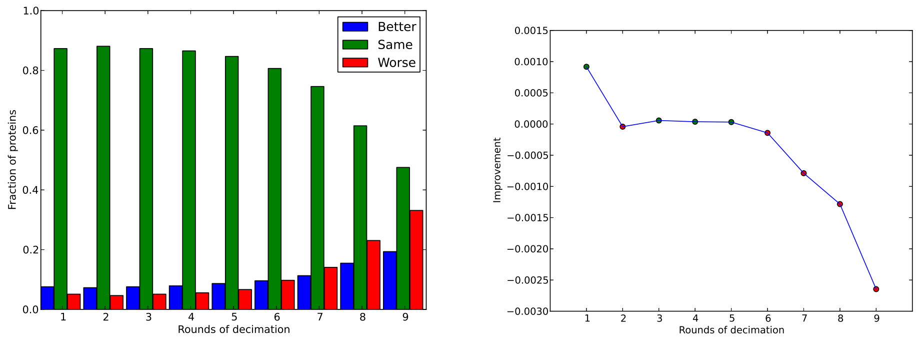

Figure 1: Effect of decimation on prediction precision. Left panel: fraction of proteins, for which decimation improves prediction in comparison to gplmDCA. Right panel: mean difference in prediction precision in comparison to gplmDCA, green dots denote mean above 0, red dots — mean below 0.
.

It appears, that the average effect of decimation on prediction accuracy is rather detrimental. While we have observed nearly no impact on prediction accuracy when considering Pfam alignments, use of decimation strategy with HHblits alignments proved to not be beneficial (c.f. Table 2 in main paper). After 9 rounds of decimation, over 35% of predictions were of lower precision than predictions without decimation and only less than 20% were of higher precision. For this data set, the greatest improvement is achieved after one round of decimation and it corresponds to average gain of 0.001 in terms of positive predictive value. This gain is equivalent to predicting one contact more accurately for each 7 proteins. As our implementation of decimation requires a full optimization for each round of decimation, we believe that currently decimation is neither feasible nor advisable for use in gplmDCA.

As we found this procedure not to improve protein contact prediction, we also implemented the decimation procedure for the symmetric version of plmDCA, minimizing an objective function of the form of Equation 1 in a setting without gap parameters. We found that neither here an improvement could be seen for a small set of test proteins (Feinauer, data not shown).

References

[1] Aurélien Decelle and Federico Ricci-Tersenghi. Pseudolikelihood decimation algorithm improving the inference of the interaction network in a general class of ising models. *Physical review letters*, 112(7):070603, 2014.

[2] Magnus Ekeberg, Tuomo Hartonen, and Erik Aurell. Fast pseudolikelihood maximization for direct-coupling analysis of protein structure from many homologous amino-acid sequences, 2014.

[3] Magnus Ekeberg, Cecilia Lövkvist, Yueheng Lan, Martin Weigt, and Erik Aurell. Improved contact prediction in proteins: Using pseudolikelihoods to infer potts models. *Physical Review E*, 87(1):012707, 2013.
